# Medicare Benefits Schedule item numbers for transcranial magnetic stimulation (TMS): Questions arising

**DOI:** 10.1177/10398562231173228

**Published:** 2023-05-02

**Authors:** Saxby Pridmore, William Pridmore

**Affiliations:** Department of Psychiatry, 3925 University of Tasmania, Hobart, TAS, Australia; 34379 Royal Hobart Hospital, Hobart, TAS, Australia

**Keywords:** Transcranial magnetic stimulation, major depressive disorder, major depressive episode, treatment-resistant depression

## Abstract

**Objective:**

Four Medicare Benefits Schedule item numbers for Transcranial Magnetic Stimulation (TMS) treatment of unresponsive MDD were declared in Australia in 2021. They are accompanied by rules/conditions. The aim is to consider these rules/conditions in light of recent research and real-world experience.

**Conclusions:**

While evidence supports some listed rules/conditions, others lack clinical justification and deserve to be reconsidered. These include (a) ineligibility of patients who have previously received TMS, (b) a lifetime total limit of 50 treatments, (c) a second/final course being unavailable for 4 months following the completion of the first course, and (d) the second/final course being limited to 15 treatments.

On 1 November 2021, four Medicare Benefits Schedule (MBS) item numbers for transcranial magnetic stimulation (TMS) treatment of treatment-resistant major depressive disorder (MDD) became operational. The applicant was the RANZCP; applications were made in 2007, 2014, 2018, and two in 2019. The Medical Services Advisory Committee (MSAC) examines evidence on safety, effectiveness, and cost-effectiveness of technologies/procedures and makes informed recommendations to Government.

TMS involves the application of an insulated metal coil to the hair/scalp and magnetic field pulses inducing changing electric fields in the underlying cortex. Depending on settings, neurons may be depolarized, and physiological activity and conductivity may be modified.^1^ TMS is a highly effective treatment of MDD.^2^ Common treatment protocols for acute MDD disorder include high frequency (10 Hz) stimulation applied to the left dorsolateral prefrontal cortex (DLPFC), and low frequency (1 Hz) simulation applied to the right DLPFC. Each treatment takes about half an hour, and a course in Australia is generally 20–30 treatments over 4–6 weeks. In the USA, up to 36 treatments have been administered in a single course.^3^

Two item numbers (14 216 and 14 217) relate to an initial treatment course, and two (14 219 and 14 220) relate to a, if required/permitted, second/final treatment course. Listed rebates are contingent upon mandatory rules/conditions.

Three rules/conditions refer to age, diagnosis, and treatment quantity. These are consistent with regulations embraced around the world and are acceptable to local service providers.

1) Patients must be at least 18 years of age.
2) Patients must have been diagnosed with MDD, and have failed to respond satisfactorily to at least two different classes of antidepressants, provided at recommended doses for recommended periods. Also, where indicated, patients must have participated and achieved insufficient benefit from psychotherapy.
3) The initial course is limited to 35 treatments.

One rule/condition applies to maintenance TMS.

4) Provision of maintenance TMS is not supported.

The MSAC has at least twice recommended against maintenance TMS (responses to Applications 1196.1 and 1196.2) based on the perception of insufficient substantiating evidence. We concede the evidence from placebo-controlled trials is not abundant. There is some recent supportive literature available,^4^ and further evidence is being assembled.^5^

Five rules/conditions appear to arise predominantly from cost-effectiveness and equity issues. These raise concerns for current local service providers, and are listed and examined below.

5) Patients who have previously received TMS are excluded from rebated treatment.
6) A lifetime maximum of two treatment courses may be provided.
7) After 50 treatments, no further treatments are available to the patient.
8) If a second/final treatment course is allowed, it is limited to 15 treatments.
9) If a second/final treatment course is allowed, it cannot commence until 4 months have transpired since the completion of the first course.

Experienced clinicians frequently reach conclusions opposite to those listed above, observing that while overservicing might be a problem with a particular patient, TMS treatment is often onerous and inconvenient for patients, and those seeking further treatments have usually benefited markedly from earlier treatment and are reasonably seeking another period of relief.

MDD may run a chronic relapsing course. More than 50% of those who experience a first episode of MDD go on to a second, and the majority experience more than two episodes.^6^ When remission is induced by ECT, for example, 37.7% relapse within 6 months.^7^ With respect to successful TMS treatment, 52.9% experience loss of response (rather than relapse) within 6 months.^8^ Research shows second and subsequent TMS courses are as effective as the first courses.^9^ Thus, as MDD is often a chronic relapsing problem, and repeat TMS courses are effective, subsequent courses are clinically justified and should be available.

Excluding rules/conditions are profoundly important to individuals who respond to TMS treatment and subsequently relapse. TMS is only available to those who have failed to respond to psychotherapy and antidepressant medication; thus, there is little likelihood of future relief via these treatments.

Where TMS treatment is no longer available (in spite of previously demonstrated efficacy), many will be forced to progress to electroconvulsive therapy (ECT). Paradoxically, there is no limit to the number of ECT which can be provided—even though ECT is more costly than TMS^10^ and less attractive to patients.^11^

The MSAC states, in response to Application 1196.3, that the limit of 15 treatments for the second/final course was derived from estimates by two USA groups of the average number of treatments usually required for a successful second course. These estimates were made a decade ago. It is time to consider the opinions of current real-world clinicians who have been working in the field for a quarter of a century. Such experts (in private discussions with the current authors) agree that while some patients (especially those who have not fully relapsed) can sometimes be restored to full remission with less than 20 treatments, most commonly, courses need to extend to 20 or more treatments.

The rule/condition that second/final TMS courses must not be provided until 4 months post the completion of the initial course is mentioned in the MSAC response to Application 1196.3. This response reports that TMS experts contend “the highest likelihood of relapse was within 3-9 months”—thus, the MSAC was advised by experts that relapse before 4 months post treatment is possible. If relapse occurs in less than 4 months, the patient’s suffering may be as great or greater than that associated with relapses which occur after 4 months—waiting for the expiration of the additional period before treatment can commence will mean additional, unnecessary suffering. Also, the longer the pathological conditions are allowed to persist before therapeutic intervention is commenced, the more difficult they may be to resolve.

## Conclusion

In November 2021, in Australia, four MBS item numbers became available for the TMS treatment of acute treatment-resistant MDD. This was an important advance in clinical care. We have calculated that when available, TMS prevents 10–15% of those presenting with major depressive episodes proceeding to ECT.^12^ Limiting the availability of TMS means treatment for some patients with a more expensive but less preferred (by the individual) method.

Thus, these item numbers are bridled by a series of rules/conditions. While many of them are appropriate and acceptable, the purpose of some appears to be the containment of costs with little regard to clinical need. For example, an embargo against those who have received TMS treatment in the past and the lifetime total limit of 50 treatments have no clinical justification and a reconsideration addressing the points raised in this paper is indicated.
